# Low expression of *bcl*-2 in *Brca1*-associated breast cancers

**DOI:** 10.1054/bjoc.2000.1438

**Published:** 2000-10-26

**Authors:** P Freneaux, D Stoppa-Lyonnet, E Mouret, M Kambouchner, A Nicolas, B Zafrani, A Vincent-Salomon, A Fourquet, H Magdelenat, X Sastre-Garau

**Affiliations:** Departments of PathologyGeneticsBiostatisticsRadiotherapyPhysiopathology, Institut Curie, 26 Rue d'Ulm, Paris cedex 05, 75248, France

**Keywords:** *Brca1*, *Brca2*, *bcl-2*, proliferation, apoptosis, breast cancer

## Abstract

Little data are available concerning the molecular mechanisms of action of *Brca1* and *Brca2* in breast oncogenesis. Recent experimental results suggest that *Brca1* plays a role in the regulation of apoptosis. In order to determine whether the analysis of human tumours would provide data supporting this hypothesis, we have assessed the expression of the antiapoptotic *bcl-2* and of the proapoptotic *p53* genes in *Brca1*- and *Brca2*-associated breast carcinomas. The levels of expression of these genes were compared to those observed in controls and to the mitotic and the apoptotic indexes. Our series were composed of 16 cases of breast carcinoma in women with a germline *Brca1* gene mutation, and of four cases with *Brca2* mutation. A group of 39 patients aged under 36 years and for whom the search for *Brca1* gene mutations was negative, and a group of 36 cases of sporadic cancers without data on their *Brca* status were used as controls. Immunohistochemistry was used to detect *p53* and *bcl-2* gene products. Mitotic and apoptotic indexes were higher in *Brca1*-associated tumours than in controls. No significant difference in p53 immunostaining was observed between the four groups of patients. In contrast, the rate of *bcl-2*-positive tumours was lower (31%) in *Brca1*-carcinomas than in carcinomas without *Brca1* mutation (90%) (*P*< 10^–3^). A strong Bcl-2 expression was found in the four cases of *Brca2*-associated carcinomas. No significant correlation was observed between p53 and Bcl-2 immunostainings, either in cases or in controls. The association between *Brca1* status and Bcl-2 expression remained significant after adjustment for the oestrogen receptor status. Our study shows that a low expression of *bcl-2* characterises most *Brca1*-associated breast carcinomas, a biological trait which seems not to be shared by *Brca2*-associated tumours nor to be related to oestrogen receptor and/or p53 status.*bcl-2* might thus be one of the target genes involved in the oncogenesis related to *Brca1* and its down-regulation may account for the increased apoptosis and the high proliferative rate observed in *Brca1*-associated carcinomas. © 2000 Cancer Research Campaign


*British Journal of Cancer* (2000) **83**(10), 1318–1322
© 2000 Cancer Research Campaign
doi: 10.1054/ bjoc.2000.1438, available online at http://www.idealibrary.com on IDEAL®

### Low expression of *bcl*-2 in *Brca1*-associated breast cancers

**P Freneaux[1], D Stoppa-Lyonnet[2], E Mouret[3], M Kambouchner[1], A Nicolas[1], B Zafrani[1], A Vincent-Salomon[1], A Fourquet[4], H Magdelenat[5] and X Sastre-Garau[1]**

[1]Departments of Pathology; [2]Genetics; [3]Biostatistics; [4]Radiotherapy; and [5]Physiopathology, Institut Curie, 26 Rue d'Ulm, 75248, Paris cedex 05, France

**Summary** Little data are available concerning the molecular mechanisms of action of *Brca1* and *Brca2* in breast oncogenesis. Recent experimental results suggest that *Brca1* plays a role in the regulation of apoptosis. In order to determine whether the analysis of human tumours would provide data supporting this hypothesis, we have assessed the expression of the antiapoptotic *bcl-2* and of the proapoptotic *p53* genes in *Brca1*- and *Brca2*-associated breast carcinomas. The levels of expression of these genes were compared to those observed in controls and to the mitotic and the apoptotic indexes. Our series were composed of 16 cases of breast carcinoma in women with a germline *Brca1* gene mutation, and of four cases with *Brca2* mutation. A group of 39 patients aged under 36 years and for whom the search for *Brca1* gene mutations was negative, and a group of 36 cases of sporadic cancers without data on their *Brca* status were used as controls. Immunohistochemistry was used to detect *p53* and *bcl-2* gene products. Mitotic and apoptotic indexes were higher in *Brca1*-associated tumours than in controls. No significant difference in p53 immunostaining was observed between the four groups of patients. In contrast, the rate of *bcl-2*-positive tumours was lower (31%) in *Brca1*-carcinomas than in carcinomas without *Brca1* mutation (90%) ($P < 10^{-3}$). A strong Bcl-2 expression was found in the four cases of *Brca2*-associated carcinomas. No significant correlation was observed between p53 and Bcl-2 immunostainings, either in cases or in controls. The association between *Brca1* status and Bcl-2 expression remained significant after adjustment for the oestrogen receptor status. Our study shows that a low expression of *bcl-2* characterises most *Brca1*-associated breast carcinomas, a biological trait which seems not to be shared by *Brca2*-associated tumours nor to be related to oestrogen receptor and/or p53 status. *bcl-2* might thus be one of the target genes involved in the oncogenesis related to *Brca1* and its down-regulation may account for the increased apoptosis and the high proliferative rate observed in *Brca1*-associated carcinomas. © 2000 Cancer Research Campaign

**Keywords:** *Brca1*; *Brca2*; *bcl-2*; proliferation; apoptosis; breast cancer

*Brca1* and *Brca2* germline mutations are frequently observed in women presenting breast carcinoma as part of an hereditary cancer susceptibility syndrome (Ford et al, 1998). The histological phenotype of *Brca*-associated breast tumours differs from that of sporadic cases: *Brca1*-associated tumours generally present as poorly differentiated and highly mitotic carcinomas (Jacquemier et al, 1995; Lakhani et al, 1998) whereas *Brca2* tumours were reported to present a high (Agnarsson et al, 1998) or a low (Lakhani et al, 1998) mitotic index. However, no characteristic immunophenotype of these lesions has been reported so far (Robson et al, 1998), and little data are available regarding the biological mechanisms involved in the development of tumours associated with *Brca1* or *2* gene mutations. Using animal models, it was recently reported that the disruption of the *Brca1* alleles specifically in mouse mammary epithelium leads to increased apoptosis and to tumour formation (Xu et al, 1999). These findings prompted us to analyse the expression of the antiapoptotic *bcl-2* and of the proapoptotic *p53* genes in a series of *Brca1*- and *Brca2*-associated breast carcinomas, together with mitotic and apoptotic indexes, and to compare these values to those obtained in sporadic cancers.

*Received 16 November 1999*
*Revised 26 June 2000*
*Accepted 29 June 2000*

*Correspondence to:* X Sastre-Garau

This analysis showed that, whereas no difference in *p53* expression was observed between the different groups, *Brca1*-associated tumours dysplayed a low rate of *bcl-2* expression and high mitotic and apoptotic indexes. The decrease in *bcl-2* expression was found to be independent from hormone receptor status and may account for the high apoptotic and proliferative rates observed in *Brca1*-associated tumours.

### MATERIAL AND METHODS

Sixteen women affected with a breast carcinoma before the age of 36 were detected to be carriers of a germline *Brca1* mutation through the *Brca1* analysis of a series of 123 early-onset consecutive cases as reported in a previous study (Ansquer et al, 1998). The corresponding 16 tumours were analysed and correspond to the group of cases. A first control group was constituted by a series of 39 women from the early-onset series identified as non-*Brca1* carriers. Tumours of 36 sporadic cases, untested for *Brca1* and *Brca2* mutation, constituted a second control group. The former group has been chosen to avoid a possible bias related to the fact that tumours occurring in young women might present a characteristic immunophenotype independently from their *Brca* status. In addition, the tumours of four women belonging to the early-onset breast carcinomas series and identified as carrier of a germline *Brca2* mutation were also studied. *p53* and *bcl-2* gene expression was assessed by immunohistochemistry using anti-p53 (D0–7) and

**Table 1** Clinicopathological characteristics of patients and tumours according to the *Brca* status

| Parameters | Cases | | Controls | | $P^a$ |
|---|---|---|---|---|---|
| | ***Brca1* mutation** | ***Brca2* mutation** | **No *Brca1* mutation** | **Sporadic tumours** | |
| Number of cases | 16 | 4 | 39 | 36 | – |
| Patient's age | | | | | |
| median | 34 | 50.5 | 33 | 62.5 | – |
| mini–maxi | 25–47 | 49–52 | 21–36 | 31–80 | |
| Tumour size | | | | | |
| ≤20 mm | 5 (31.2%) | 2 (50%) | 17 (43.6%) | 9 (25%) | |
| >20 mm | 11 (68.8%) | 2 (50%) | 22 (56.4%) | 27 (75%) | ns |
| Node status | | | | | |
| N0 | 6 (37.5%) | 3 (75%) | 26 (66.7%) | 26 (72.2%) | |
| N1 | 10 (62.5%) | 0 | 13 (33.3%) | 9 (25%) | $4.6 \times 10^{-2}$ |
| N2 | 0 | 1 (25%) | 0 | 1 (2.8%) | |
| Histological grade | | | | | |
| I | 0 | 0 | 3 (7.7%) | 7 (19.4%) | |
| II | 4 (25%) | 1 (25%) | 27 (69.2%) | 16 (44.4%) | $3.10^{-4}$ |
| III | 12 (75%) | 3 (75%) | 9 (23.1%) | 13 (36.2%) | |
| Mitotic index[b] | 14.5 | 5.5 | 4 | 8 | $9.10^{-3}$ |
| Apoptotic index[b] | 10 | 9 | 7 | nd | $2.10^{-2}$ |
| Hormonal status | | | | | |
| ER-positive | 3 (21.4%) | 4 (100%) | 27 (71.1%) | 26 (74.3%) | $10^{-3}$ |
| PR-positive | 7 (46.7%) | 3 (75%) | 28 (73.7%) | 23 (65.7%) | ns |
| nd | 3 | | 1 | 1 | |
| Bcl-2 status | | | | | |
| positive cases | 5 (31%) | 4 (100%) | 35 (90%) | 27 (77%) | $<10^{-3c}$ |
| P53 status | | | | | |
| positive cases | 9 (56.2%) | 0 | 24 (61.5%) | 13 (38.2%) | ns |
| nd | | | | 2 | |

[a]*Brca1* cases vs no *Brca1* mutation; [b]median of indexes; [c]Chi-squared test with Yate's correction; nd = not determined; ns = not significant anti-Bcl-2 (124) monoclonal antibodies (Dako A/S, Glostrup, Denmark). The threshold of positivity was 5% of malignant stained cells for p53 and 1% for Bcl-2. Proliferation rate was determined by counting the number of mitotic features in ten successive microscopic fields at G × 400, as recommended for the evaluation of the Nottingham histological grade (Elston and Ellis, 1991; Genestie et al, 1998). Apoptotic index was assessed on haematoxylin–eosin-coloured histological sections by counting nuclear and/or cytoplasmic features characterising the apoptotic process (Bessis, 1964; Cummings et al, 1997). In every case, a tumour area presenting the highest number of apoptotic features was determined at low magnification, then analysed at high power field (G × 400). After image capture, apoptotic features were pointed on the screen and scored using an image analysis system (Perfect Image; Clara Vision, Orsay, France). The apoptotic index was defined as the ratio between this count and the total number of invasive malignant cells. The counting was performed on successive fields when less than 100 invasive malignant cells were present on a single field.

The association between *Brca1* gene mutation and tumour characteristics was evaluated using the Chi-squared test with Yate's correction when necessary for discrete variables (tumour size, nodal status, histological grade, hormonal status, *bcl-2* status, *p53* status) and the Mann–Whitney test for continuous variables (age, mitotic index, apoptotic index). Logistic regression was used to assess the independent association of factors for *Brca1* gene mutation identified in univariate analysis, in a group of tumours for which all clinical and pathological data were available (52 tumours), i.e. 13 cases with *Brca1* mutation and the 39 controls with no *Brca1* mutation. All variables with $P$ value < 0.05 were introduced in the initial multivariate model (excepted mitotic and apoptotic indexes) and deleted through a backward procedure. Variables with a significance level of 0.05 were kept in the final model.

### RESULTS

Our analysis showed that a higher histological grade ($P = 3.10^{-4}$), a higher mitotic index ($P = 9.10^{-3}$), a higher apoptotic index ($P = 2.10^{-2}$) and a lower rate of oestrogen receptor positivity ($P = 10^{-3}$) were observed in *Brca1*-associated breast cancers than in controls (Table 1). A slight positive correlation was found between mitotic and apoptotic indexes ($r = 0.28$, $P = 0.045$).

The immunohistochemical analysis found no significant difference in p53 immunostaining between the four groups of tumours. In contrast, a significant difference in *bcl-2* expression was found between cases and either control groups (Figure 1). The rate of *bcl-2*-positive tumours was lower (31%) in *Brca1*-associated carcinomas than in sporadic tumours (77%) ($P = 2.10^{-3}$). This difference was even more striking in comparison with the rate of *bcl-2*-positive tumours observed in young women without *Brca1* mutation (90%) ($P < 10^{-3}$). The four cases of *Brca2*-associated carcinoma were found to be positive. The mean number of labelled cells was 56% in the group of sporadic tumours and 45% in tumours with no *Brca1* mutation. In contrast, a complete absence of labeling was observed in 11 of the 16 cases of *Brca1*-associated carcinomas. A weak staining of 5% of the tumour cells was observed in one case whereas the remaining four cases exhibited 40–80% of labelled cells. The negative association of *bcl-2* expression and *Brca1*-mutation remained significant after

© 2000 Cancer Research Campaign

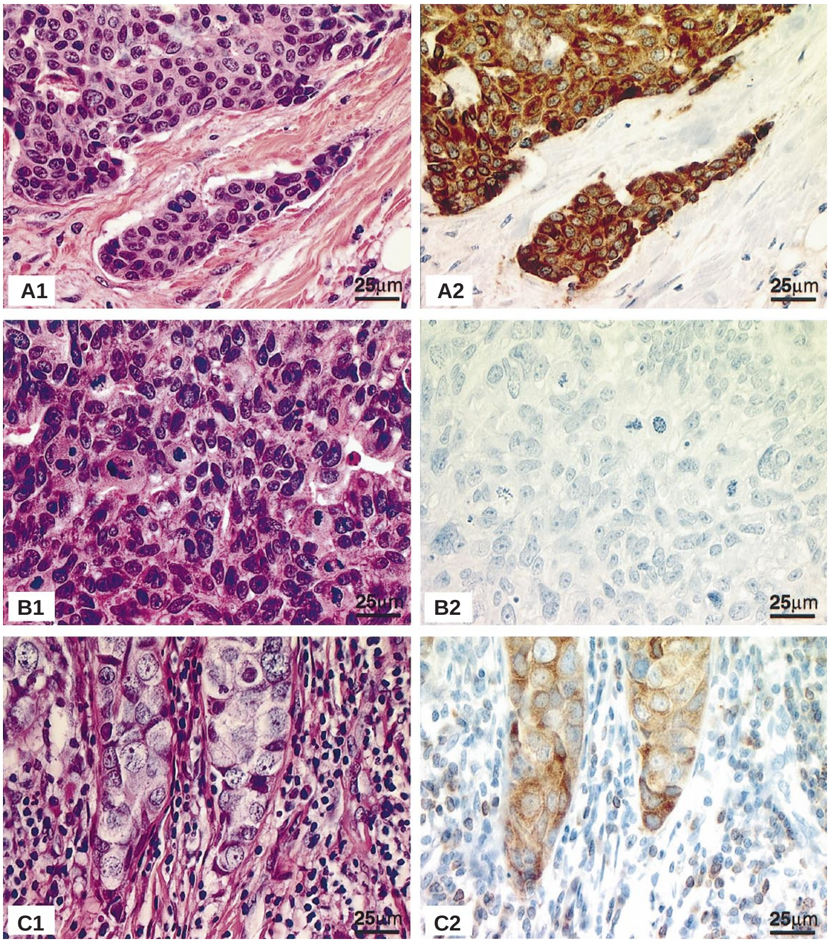

**Figure 1** Histological aspects of sporadic invasive ductal carcinoma (**A1**), *Brca1*-associated (**B1**) and *Brca2*-associated (**C1**) carcinomas. Immunostainings of *bcl-2* in these cases. High level of *bcl-2* expression in sporadic cancer (**A2**) and in *Brca2*-associated carcinoma (**C2**). No expression detected in *Brca1*-associated carcinoma (**B2**)

adjustment on oestrogen receptor status. In multivariate logistic regression analysis, low expression of *bcl-2* (odds ratio (OR) 13.9; 95% CI 2.6–76.9) and high histological grade (OR 7.1; 95% CI 1.3–38.8) were significant predictive variables associated with the presence of *Brca1* gene mutation (Table 2). No significant correlation was observed between p53 and Bcl-2 immunostaining, either in cases or in controls.

### DISCUSSION

Our study shows that most *Brca1*-associated breast carcinomas are characterized by high mitotic and apoptotic rates, and by a decrease of *bcl-2* expression. These biological traits seem not to be shared by *Brca2*-associated tumours and not related to oestrogen receptor and/or to p53 status. In an analysis of the immunophenotype of *Brca*-associated tumours, only a slight decrease of *bcl-2* expression was found in familial cancers in comparison with the level observed in controls (Robson et al, 1998). However, in this work, *Brca1*- and *Brca2*-tumours were included in the same group, and this could account for the negativity of the result. Recent data emphasize that different molecular pathogenetic processes characterize *Brca1*- and *Brca2*-associated tumours (Armes et al, 1999), and this is in agreement with our data on distinctive *bcl-2*-expression in these two tumour types.

High levels of *bcl-2* expression have been observed in 75–80% of cases of sporadic breast cancers (Leek et al, 1994; Silvestrini et al, 1994; Hellemans et al, 1995). This expression was found to be associated with the presence of favourable prognostic features (Joensuu et al, 1994) and with the expression of oestrogen and progesterone receptors (Barghava et al, 1994; Gee et al, 1994; Leek et al, 1994; Silvestrini et al, 1994; Hellemans et al, 1995; Charpin et al, 1997).

Since *Brca1*-associated breast carcinomas are frequently oestrogen receptor negative, poorly differentiated tumours, and since a positive association exists between the respective expression of oestrogen receptor (ER) and *bcl-2*, the down-regulation of *bcl-2* in *Brca1*-associated carcinomas might merely reflect the down regulation of ER in this type of tumour. In our study, the loss of *bcl-2* expression characterizing *Brca1*-associated carcinomas behaved as a biological trait independent from the ER status of the tumours. Definitive conclusions cannot be drawn from this multivariate analysis considering the limited number of cases included in this series. However, it is worth mentioning that in multivariate analyses of prognostic factors characterizing large series of axillary node-positive breast tumours, the lack of *bcl-2* expression was found associated with a poor outcome of the disease, independently from the ER status (Hellemans et al, 1995; Berardo et al, 1998).

 © 2000 Cancer Research Campaign

**Table 2** Association of biological parameters with *Brca1* gene mutation in logistic regression model[a]

| Biological parameters | OR | 95% CI | *P* value |
|---|---|---|---|
| *Bcl-2* expression | | | |
| high | 1 | | |
| low | 13.9 | 2.6–76.9 | 0.002 |
| histological grade | | | |
| I/II | 1 | | |
| III | 7.1 | 1.3–38.8 | 0.02 |

OR = adjusted odds ratio; CI = confidence interval; [a]*Brca1* mutated and not mutated tumours

The molecular mechanisms of action of *Brca1* in the tumour process are poorly understood. The observation of increased apoptosis in mouse mammary tissue triggered by the inactivation of the two copies of the *Brca1* gene (Xu et al, 1999) suggest that the *Brca1* gene products could function as transcriptional regulators of (Yang and Lippman, 1999). The anti-apoptotic *bcl-2* might be one of the target genes for *Brca1* in such mechanisms. Our present data reporting a down-regulation of *bcl-2* and a high apoptotic index of *Brca1*-associated carcinomas support this hypothesis. Another line of evidence indicates that *Brca1* plays a positive role in the control of cell growth (Gudas et al, 1996). *bcl-2* gene product could also act as a second messenger in this control: an inhibitory effect of entry into the cell cycle has been reported for *bcl-2* (O'Reilly et al, 1996). and this effect was found genetically separable from the functions of the protein in cell survival (Huang et al, 1997). The high proliferative rate observed in *Brca1*-, but not in *Brca2*-associated tumours (Armes et al, 1999), might thus be related to the respective levels of *bcl-2* expression in these tumours. It is worth mentioning that a positive link between proliferation and apoptosis has already been observed in carcinomas of the breast (Dowsett et al, 1999), and that the loss of *bcl-2* has been found to be associated with both increased apoptosis and increased proliferative rates in sporadic breast cancers (van Slooten et al, 1998). In such cases, it would be of interest to determine whether this loss was associated with a down-regulation of *Brca1*, the implication of which in the progression of sporadic breast carcinomas is also a matter of speculation (Yang and Lippman, 1999).

The similarity in p53 immunostaining between *Brca1*-and non *Brca1*-associated tumours is in agreement with our previous results reporting a similar pattern in *p53* mutations in these two groups of tumours (Schlichtholz et al, 1998). These results are at discrepancy with data reporting a high rate of p53 mutation in a small group of *Brca1*-associated carcinomas (Crook et al, 1997). It is worth mentioning that a very large diversity of p53 mutational patterns is observed among cohorts of sporadic breast cancers (Hartmann et al, 1997). These variations may also concern familial tumours and thus account, at least in part, for the discrepancies observed between the rates of p53 alterations in these tumours. Furthermore, the biological consequence of p53 cell accumulation, as determined by immunohistochemistry, is unclear and the link between p53-positive immunostaining and gene mutation is inconstant in breast cancers (Sjögren et al, 1996). No correlation was observed between *p53* and *bcl-2* immunostaining in our study, a result already reported by others (Silvestrini et al, 1996).

In conclusion, a low *bcl-2* expression and high proliferative and apoptotic rates characterize most *Brca1*-associated breast carcinomas. The analysis of a large series of cases including the assessment of the expression of *bcl-2*, of *Brca1* and of biological parameters associated with cell death and proliferation, should help to determine whether alterations of the functions of *Brca1* gene product were responsible for the down-regulation of *bcl-2*, and thus for the high proliferative rate and increased apoptosis observed in *Brca1*-associated carcinomas.

### ACKNOWLEDGEMENTS

We thank Dr Jérôme Couturier for helpful discussion. This work was supported in part by a grant from the Lion's Club (Moulins/s/Allier).

### REFERENCES

Agnarsson B, Jonasson J, Bjornsdottir I, Barkardottir R, Egilsson V and Sigurdsson H (1998) Inherited BRCA2 mutation associated with high grade breast cancer. *Breast Cancer Res Treat* **47**: 121–127

Ansquer Y, Gautier C, Fourquet A, Asselain B, Stoppa-Lyonnet D and the Institut Curie Breast Cancer Group (1998) Survival in early-onset BRCA1-cancer patients. *Lancet* **352**: 541

Armes J, Trute L, White D, Southey C, Hammet F, Tesoriero A, Hutchins A, Dite G, McCredie M, Giles G, Hopper J and Venter D (1999) Distinct molecular pathogeneses of early-onset breast cancers in *BRCA1* and *BRCA2* mutation carriers: a population-based study. *Cancer Res* **59**: 2011–2017

Barghava V, Kell D, van de Rijn M and Warnke R (1994) *Bcl-2* immunoreactivity in breast carcinoma correlates with hormone receptor positivity. *Am J Pathol* **145**: 535–540

Berardo M, Elledge R, de Moor C, Clark G, Osborne K and Allred C (1998) *bcl-2* and apoptosis in lymph node positive breast carcinoma. *Cancer* **82**: 1296–1302

Bessis M (1964) Studies on cell agony and death: an attempt at classification. In *Ciba Foundation Symposium on Cellular Injury* AVS de Reuck and J Knight (eds). J & A Churchill Ltd: London

Charpin C, Garcia S, Bouvier C, Devictor B, Andrac L, Lavaut M.-N and Allasia C (1997) Automated and quantitative immunocytochemical assays of Bcl-2 protein in breast carcinomas. *Br J Cancer* **76**: 340–346

Crook T, Crossland S, Crompton M, Osin P and Gusterson B (1997) p53 mutations in BRCA 1-associated familiar breast cancer. *Lancet* **350**: 638–639

Cummings M, Winterford C and Walker N (1997) Apoptosis. *Am J Surg Pathol* **21**: 88–101

Dowsett M, Smith I, Powles T, Salter J, Ellis P, Johnston S, Makris A, Mainwaring P, Gregory R, Archer C, Chang J and Assersohn L (1999) Biological studies in primary medical therapy of breast cancer: The Royal Marsden Hospital experience. In *ESO Scientific Updates, Vol 4. Primary Medical Therapy for Breast Cancer: Clinical and Biological Aspects*, A. Howell and M. Dowsett (eds) pp 113–125. Elsevier Science: London

Elston C and Ellis I (1991) Pathological prognostic factors in breast cancer. I. The value of histological grade in breast cancer: experience from a large study with long-term follow-up. *Histopathol* **19**: 403–410

Ford D, Easton D, Stratton M, Narod S, Goldgar D, Devilee P, Bishop D, Weber B, Lenoir G, Chang-Claude J, Sobol H, Teare M, Struewing J, Arason A, Scherneck S, Peto J, Rebbeck T, Tonin P, Neuhausen S, Barkardottir R, Eyfjord J, Lynch H, Ponder B, Gayther S, Birch J, Lindblom A, Stoppa-Lyonnet D, Bignon Y, Borg A, Hamann U, Haites N, Scott R, Maugard C, Vasen H, Seitz S, Cannon-Albright L, Schofield A, Zelada-Hedman M and Consortium BCL (1998) Genetic heterogeneity and penetrance analysis of the BRCA1 and BRCA2 genes in breast cancer families. *Am J Hum Genet* **62**: 676–689

Gee J, Robertson J, Ellis I, Willsher P, McClelland R, Hoyle H, Kyme S, Finlay P, Blamey R and Nicholson R (1994) Immunocytochemical localization of BCL-2 protein in human breast cancers and its relationship to a series of prognostic markers and response to endocrine therapy. *Intl J Cancer* **59**: 619–628

Genestie C, Zafrani B, Asselin B, Fourquet A, Rozan S, Validire P, Vincent-Salomon A and Sastre-Garau X (1998) Comparison of the respective prognostic values of Scarff-Bloom-Richardson and Nottingham histological grade in a series of 825 cases of breast cancer. Major importance of the mitotic count as a

© 2000 Cancer Research Campaign 

component of both grading systems. *Anticancer Res* **18**: 571–576
Gudas J, Li T, Nguyen H, Jensen D, Rauscher F and Cowan K (1996) Cell cycle regulation of *BRCA1* messenger RNA in human breast epithelial cells. *Cell Growth Differ* **7**: 717–723
Hartmann A, Blaszyk H, Kovach J and Sommer S (1997) The molecular epidemiology of P53 gene mutations in human breast cancer. *Trends Genet* **13**: 27–33
Hellemans P, van Dam P, Weyler J, van Oosterom A, Buytaert P and Van Marck E (1995) Prognostic value of *bcl-2* expression in invasive breast cancer. *Br J Cancer* **72**: 354–360
Huang D, O'Reilly L, Strasser A and Cory S (1997) The anti-apoptosis function of Bcl-2 can be genetically separated from its inhibitory effect on cell cycle entry. *EMBO J* **16**: 4628–4638
Jacquemier J, Eisinger F, Birnbaum D and Sobol H (1995) Histoprognostic grade in *BRCA1*-associated breast cancer. *Lancet* **345**: 1503
Joensuu H, Pylkkänen L and Toikkanen S (1994) Bcl-2 protein expression and long-term survival in breast cancer. *Am J Pathol* **145**: 1191–1198
Lakhani S, Jacquemier J, Sloane J, Gusterson B, Anderson T, van de Vijver M, Farid L, Venter D, Antoniou A, Storfer-Isser A, Smyth E, Steel M, Haites N, Scott R, Goldgar D, Neuhausen S, Daly P, Ormiston W, McManus R, Scherneck S, Ponder B, Ford D, Peto J, Stoppa-Lyonnet D, Bignon Y, Struewing J, Spurr N, Bishop D, Devilee P, Cornelisse C, Lasset C, Lenoir G, Bjork Barkardottir R, Egilsson V, Hamann U, Chang-Claude J, Sobol H, Weber B, Stratton M and Easton D (1998) Multifactorial analysis of differences between sporadic breast cancers and cancers involving BRCA1 and BRCA2 mutations. *J Natl Cancer Inst* **90**: 1338–1345
Leek R, Kaklamanis L, Pezzella F, Gatter K and Harris A (1994) *bcl-2* in normal breast and carcinoma, association with oestrogen receptor-positive, epidermal growth factor receptor-negative tumours and *in situ* cancer. *Br J Cancer* **69**: 135–139
O'Reilly L, Huang D and Strasser A (1996) The cell death inhibitor Bcl-2 and its homologues influence control of cell cycle entry. *EMBO J* **15**: 6979–6990
Robson M, Rajan P, Rosen P, Gilewski T, Hirschaut Y, Pressman P, Haas B, Norton L and Offit K (1998) BRCA-associated breast cancer: absence of a characteristic immunophenotype. *Cancer Res* **58**: 1389–1842
Schlichtholz B, Bouchind'homme B, Pages S, Martin E, Liva S, Magdelenat H, Sastre-Garau X, Stoppa-Lyonnet D and Soussi T (1998) p53 mutations in BRCA1-associated familial breast cancer. *Lancet* **352**: 622
Silvestrini R, Benini E, Veneroni S, Daidone M, Tomasic G, Squicciarini P and Salvadori B (1996) p53 and bcl-2 expression correlates with clinical outcome in a series of node-positive breast cancer patients. *J Clin Oncol* **14**: 1604–1610
Silvestrini R, Veneroni S, Daidone M, Benini E, Boracchi P, Mezzetti M, Di Fronzo G, Rilke F and Veronesi U (1994) The Bcl-2 protein: a prognostic indicator strongly related to p53 protein in lymph node-negative breast cancer patients. *J Natl Cancer Inst* **86**: 499–504
Sjögren S, Inganäs M, Norberg T, Lindgren A, Nordgren H, Holmberg L and Bergh J (1996) The p53 gene in breast cancer: prognostic value of complementary DNA sequencing versus immunohistochemistry. *J Natl Cancer Inst* **88**: 173–182
van Slooten H.-J, van de Vijver M, van de Velde C and van Dierendonck J (1998) Loss of Bcl-2 in invasive breast cancer is associated with high rates of cell death, by also with increased proliferative activity. *Br J Cancer* **77**: 789–796
Xu X, Wagner K, Larson D, Weaver Z, Li C, Ried T, Hennighausen L, Wynshaw-Boris A and Deng C (1999) Conditional mutation of Brcal in mammary epithelial cells results in blunted ductal morphogenesis and tumour formation. *Nature Genet* **22**: 37–43
Yang X and Lippman M (1999) *BRCA1* and *BRCA2* in breast cancer. *Breast Cancer Res Treat* **54**: 1–10

 *© 2000 Cancer Research Campaign*
